# Complication patterns in patients undergoing venoarterial extracorporeal membrane oxygenation in intensive care unit: Multiple correspondence analysis and hierarchical ascendant classification

**DOI:** 10.1371/journal.pone.0203643

**Published:** 2018-09-11

**Authors:** Jérôme Allyn, Cyril Ferdynus, Hugo Lo Pinto, Bruno Bouchet, Romain Persichini, David Vandroux, Berenice Puech, Nicolas Allou

**Affiliations:** 1 Réanimation Polyvalente, Centre Hospitalier Universitaire de La Réunion Site Félix Guyon, Saint-Denis, France; 2 Departement d’Informatique Clinique, Centre Hospitalier Universitaire de La Réunion Site Félix Guyon, Saint-Denis, France; 3 Unité de Soutien Méthodologique, Centre Hospitalier Universitaire de La Réunion, Saint-Denis, France; 4 INSERM, CIC 1410, Saint-Pierre, France; IRCCS Policlinico S.Donato, ITALY

## Abstract

**Background:**

Treatment by venoarterial extracorporeal membrane oxygenation (VA-ECMO) is widely used today, even though it is associated with high risks of complications and death. While studies have focused on the relationship between some of these complications and the risk of death, the relationship between different complications has never been specifically examined, despite the fact that the occurrence of one complication is known to favor the occurrence of others. Our objective was to describe the relationship between complications in patients undergoing VA-ECMO in intensive care unit (ICU) and to identify, if possible, patterns of patients according to complications.

**Methods and findings:**

As part of a retrospective cohort study, we conducted a multiple correspondence analysis followed by a hierarchical ascendant classification in order to identify patterns of patients according to main complications (sepsis, thromboembolic event, major transfusion, major bleeding, renal replacement therapy) and in-ICU death. Our cohort of 145 patients presented an in-ICU mortality rate of 50.3%. Morbidity was high, with 36.5% of patients presenting three or more of the five complications studied. Multiple correspondence analysis revealed a cumulative inertia of 76.9% for the first three dimensions. Complications were clustered together and clustered close to death, prompting the identification of four patterns of patients according to complications, including one with no complications.

**Conclusions:**

Our study, based on a large cohort of patients undergoing VA-ECMO in ICU and presenting a mortality rate comparable to that reported in the literature, identified numerous and often interrelated complications. Multiple correspondence analysis and hierarchical ascendant classification yielded clusters of patients and highlighted specific links between some of the complications studied. Further research should be conducted in this area.

## Introduction

Management of the most severe patients in intensive care unit (ICU) often requires the use of venoarterial (VA) or venovenous (VV) extracorporeal membrane oxygenation (ECMO). The most common indications are acute respiratory distress syndrome (ARDS) for VV-ECMO and cardiogenic shock and postoperative cardiac surgery for VA-ECMO. However, these widely used techniques are associated with high risks of complications and death, the mortality rate being around 40% for VV-ECMO and 55% for VA-ECMO [1–3]. The most frequent complications are hemorrhagic, thrombotic, and infectious events [3–7]. While previous studies have focused on the relationship between some of these complications and the risk of death, the relationship between different complications has never been specifically examined, despite the fact that the occurrence of one complication is known to favor the occurrence of others [1–3,8,9]. For example, hemorrhagic shock and/or blood transfusion can favor the occurrence of a bacterial infection and/or of a thrombotic event [10–12].

Describing the relationship between different types of complications is of interest for various reasons. First, it allows for a better understanding of the pathophysiology of complications. Second, it helps to identify patterns of patients who might benefit from specific care. Such analysis of the relationship between different variables has already been conducted in other fields [13–16].

Our objective was to describe the relationship between complications in patients undergoing VA-ECMO in ICU and to identify, if possible, patterns of patients according to complications.

## Methods

### Study design and data collection

Data from an observational cohort study known as the ECMO-CGR study was retrospectively analyzed [17] (S1 Table). ECMO-CGR study was conducted from January 2010 to December 2016 in a single center: a French university hospital with a 23-bed mixed medical/surgical ICU and a cardiac surgery unit. All patients hospitalized in ICU who underwent ECMO during their stay were consecutively included. The exclusion criteria in the present study were age below 18 years, treatment by VV-ECMO, and ECMO duration less than two days.

This present study was approved by the Ethical Committee of the French Intensive Care Society, which waived the need for informed consent because of the observational and retrospective nature of the study (reference SRLF “CE-SRLF17-18”). Reporting of this study complied with the Strengthening the Reporting of Observational studies in Epidemiology recommendations [18].

### Definitions

Thrombotic event was defined as transient ischemic attack, ischemic stroke, systemic embolism, acute myocardial infarction, intra-cardiac thrombosis, deep venous thrombosis, pulmonary embolism, and circuit/membrane oxygenator change due to thrombus formation.

Major bleeding was defined according to the criteria of the Extracorporeal Life Support Organization as bleeding responsible for death, intracranial bleeding, bleeding requiring hemostatic procedure (surgery, embolization, or gastroscopy), or bleeding associated with administration ≥ 2 red blood cell (RBC) units in 24 hours [19].

Major transfusion was defined as administration of RBC units per day of hospitalization in ICU greater ≥ 2.

Sepsis was defined as the occurrence of a documented nosocomial infection (bacteremia, pneumonia, surgical site infection and/or catheter-related infection) or the occurrence of septic shock [20].

Use of renal replacement therapy (regardless of prior status) and in-ICU mortality were also investigated.

### Statistical analysis

Results were expressed as total number (percentage) for categorical variables and as median [25^th^;75th percentiles] for continuous variables, as appropriate. Continuous variables were compared using the Mann–Whitney U test, and categorical variables were compared using the χ2 test, as appropriate. The complications selected (sepsis, thromboembolic event, major bleeding, major transfusion, and renal replacement therapy) and the in-ICU mortality rate were coded into two modalities (presence or absence). A multiple correspondence analysis and a hierarchical ascendant classification aimed at identifying patterns according to complications (“death” was coded as an illustrative variable) were performed using R software version 3.2.2 (The R Foundation for Statistical Computing; Vienna, Austria), with packages FactoMineR [21]. Multiple correspondence analysis is a form of vector analysis whereby a set of individuals is described via a set of qualitative variables. It is used to represent individuals in a multidimensional space, the axes of which do not constitute variables but are recalculated to concentrate the majority of variations [22]. Our multiple correspondence analysis revealed: *i*) cumulative inertia for n = x dimensions, which corresponds to the variation observed in the cohort for n = x dimensions; and *ii*) figures that depict the relationship between two dimensions for variables and for patients (for example, between the first and second dimensions or between the first and third dimensions).

Hierarchical ascendant classification is used to partition a population into subgroups via a distance matrix. It serves to create a tree diagram known as dendrogram, which illustrates the arrangement of clusters.

## Results

### Characteristics of patients

Over the study period, 242 patients underwent ECMO. Among these, 97 patients were excluded (32 received ECMO for less than two days, 9 were less than 18 years old, and 56 received VV-ECMO). The remaining 145 patients constituted the cohort.

The median age was 56 [44;64] and the median Simplified Acute Physiology Score (SAPS) II was 56 [43;72]. The main indications for VA-ECMO were postcardiotomy cardiogenic shock in 56 cases (38.6%), acute myocardial infarction in 47 cases (32.4%), refractory cardiac arrest in 16 cases (11%), dilated cardiomyopathy in 10 cases (6.9%), ARDS in 9 cases (6.2%), and myocarditis in 7 cases (4.8%). The median duration of ECMO was 7 [4;11] days.

### Complications and prognosis

The in-ICU mortality rate was 50.3%. The median length of stay in ICU and in hospital were 14 [9;21] days and 21 [11;37] days, respectively. Patients received 1 [0.55;1.75] RBC unit per day of ECMO. Table 1 summarizes the frequencies of the complications studied and their relationship with death. Among these complications (except death), major bleeding and renal replacement therapy were found to be significantly associated with fatal outcome in bivariate analysis (*p =* 0.046 and *p*<0.001, respectively). Overall, 15.9%, 22.1%, 25.6%, 24.1%, 12.4% of patients presented, respectively, zero, one, two, three, and more than three of the five complications studied (all variables except death, *i*.*e*., sepsis, major transfusion, thrombotic event, major bleeding, and renal replacement therapy). Moreover, we found significantly more complications per patient in the group of deceased patients than in the group of patients who survived (3 [2;3] *versus* 1 [0.75;2], *p*<0.001).

**Table 1:** Complications from ECMO for total population, deceased patients, and surviving patients. The groups “Deceased” and “Surviving” were compared using the χ2 test. ECMO: extracorporeal membrane oxygenation.

Complication from ECMO	Total (n = 145)	Deceased (n = 99)	Surviving (n = 102)	*p*
Major transfusion, n (%)	32 (22.1)	21 (14.5)	11 (7.6)	0.07
Thrombotic event, n (%)	35 (24.1)	18 (12.4)	17 (11.7)	1
Major bleeding, n (%)	69 (47.6)	41 (28.3)	28 (19.3)	0.046
Sepsis, n (%)	65 (44.8)	38 (26.2)	27 (18.6)	0.096
Renal replacement therapy, n (%)	83 (57.2)	57 (39.3)	26 (17.9)	<0.001

#### Multiple correspondence analysis and hierarchical ascending classification

Multiple correspondence analysis revealed a cumulative inertia of 76.9% for the first three dimensions, meaning that the first three dimensions accounted for 76.9% of the variations observed in the cohort. The first-dimension inertia was 31.5%, the second was 26.2%, and the third was 19.3%.

**Fig 1:**
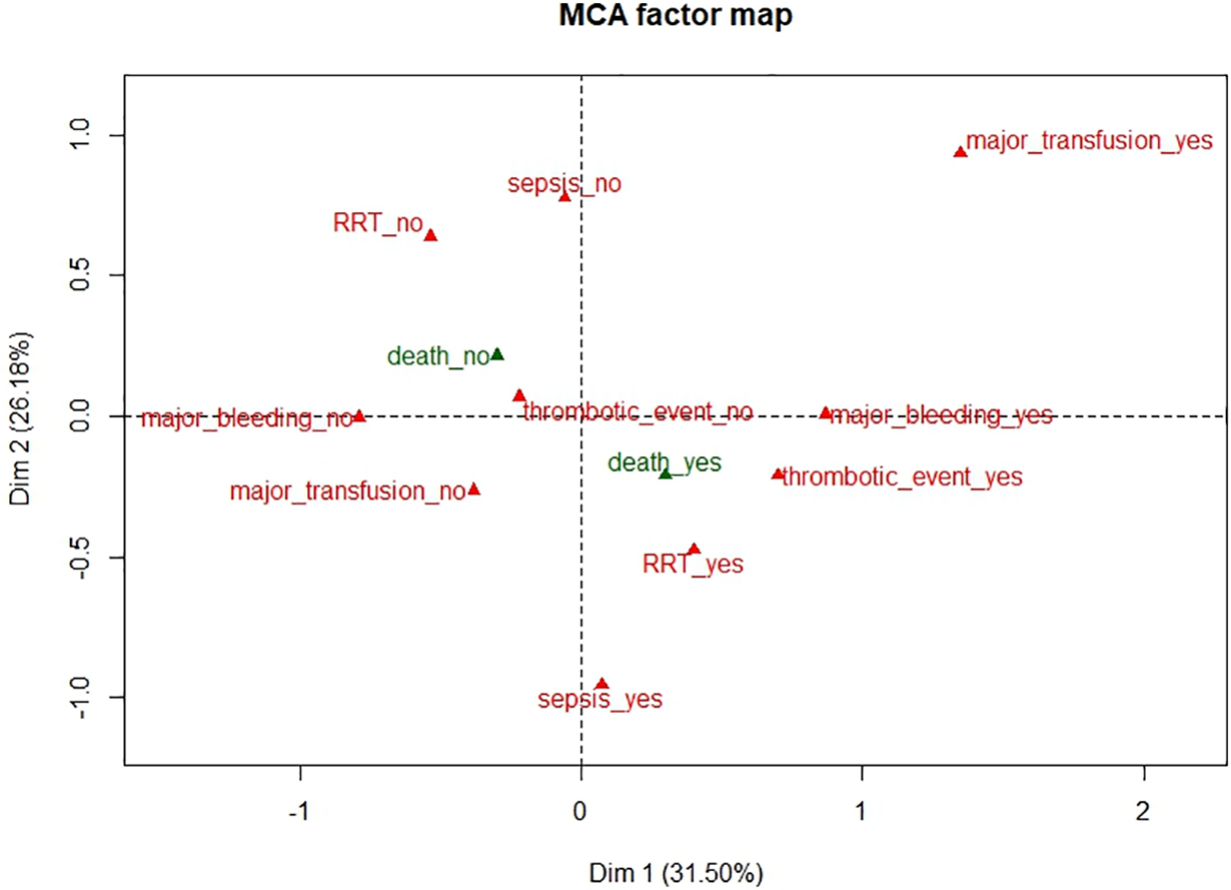
Multiple correspondence analysis: Variable plot for dimensions 1 and 2. Active modalities are in red and illustrative modalities are in green. RRT: Renal replacement therapy. This figure represents the two-dimensional (dimensions 1 and 2) graph generated from the estimated model, which showed the importance of the different complication variables and their impact on data distribution.

**Fig 2:**
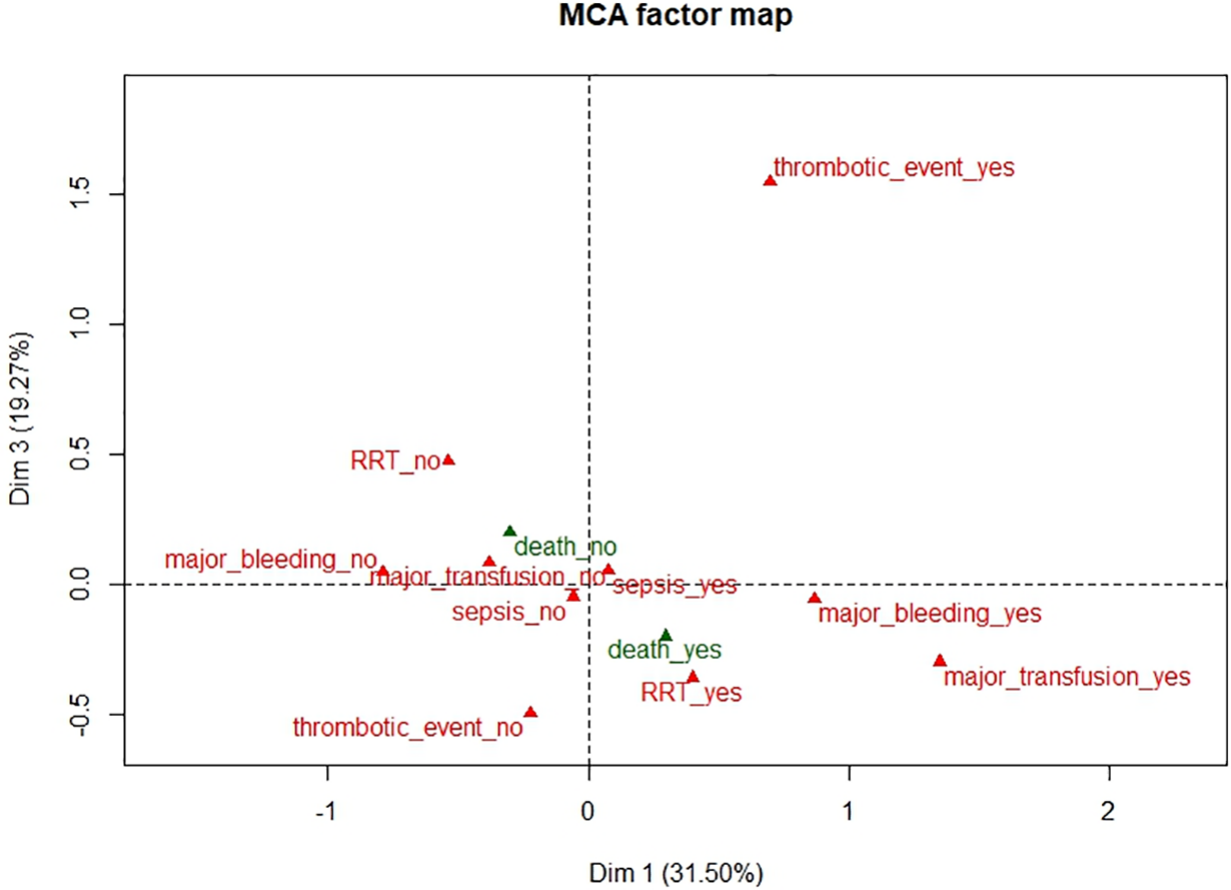
Multiple correspondence analysis: Variable plot for dimensions 1 and 3. Active modalities are in red and illustrative modalities are in green. RRT: Renal replacement therapy. This figure represents the two-dimensional (dimensions 1 and 3) graph generated from the estimated model, which showed the importance of the different complication variables and their impact on data distribution.

Figs 1 and 2 depict the relationship between the first and second dimensions for variables and for patients, respectively.

In the first dimension, individuals with major bleeding, major transfusion, or thrombotic event (on the right of the graph) are mostly located opposite individuals without these complications (on the left of the graph). Similarly, in the second dimension, individuals with major transfusion are located opposite individuals with sepsis or renal replacement therapy. The third dimension depends mainly on the presence of a thrombotic event.

**Fig 3:**
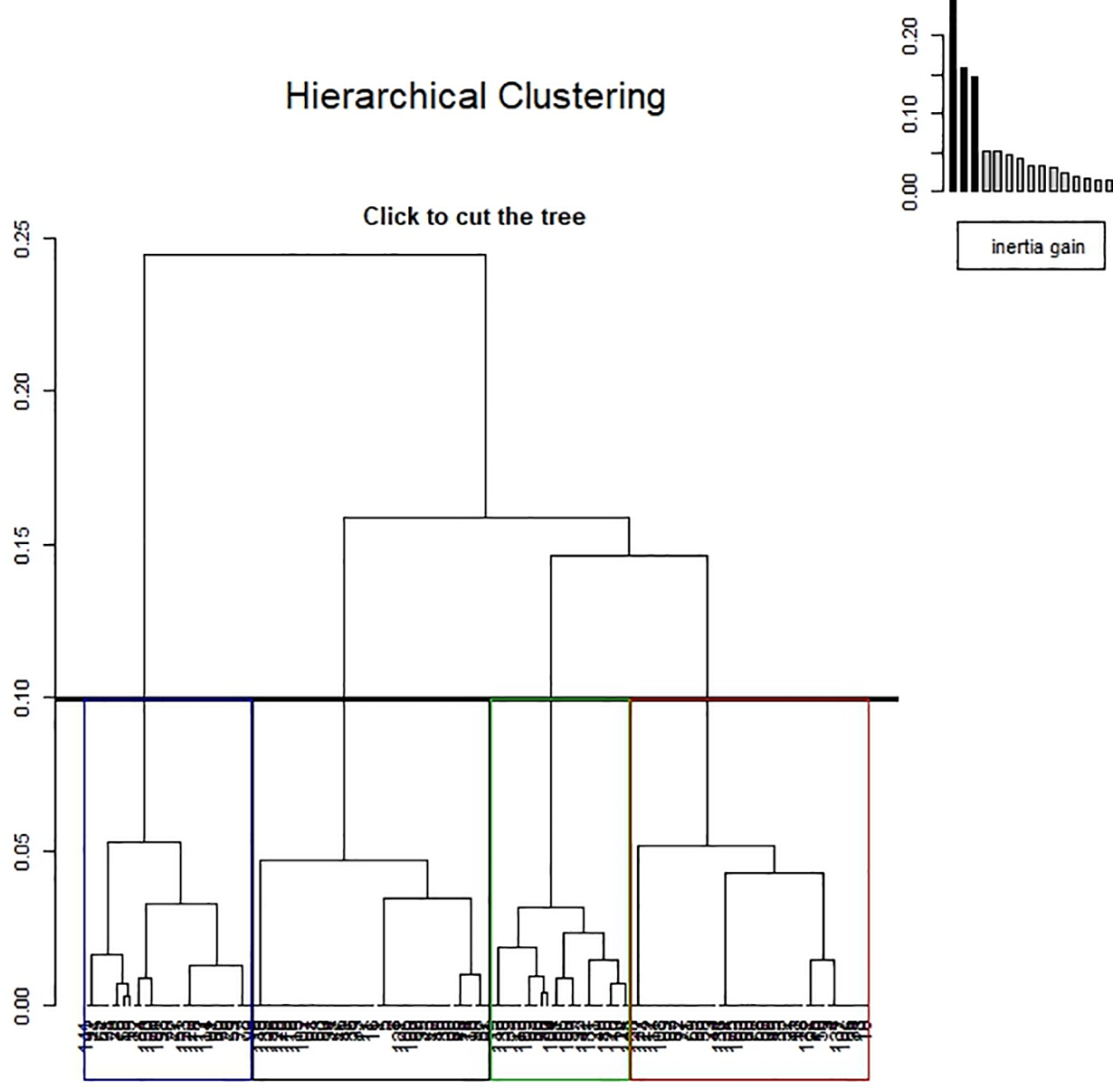
Hierarchical ascending classification of patients: Dendrogram. A dendrogram is a tree diagram which is used to illustrate the arrangement of clusters. Here, individuals are plotted on the abscissa axis. The black, red, green, and blue rectangles define the four clusters of patients. The graph in the top right corner represents the loss of inertia according to the different dimensions.

**Fig 4:**
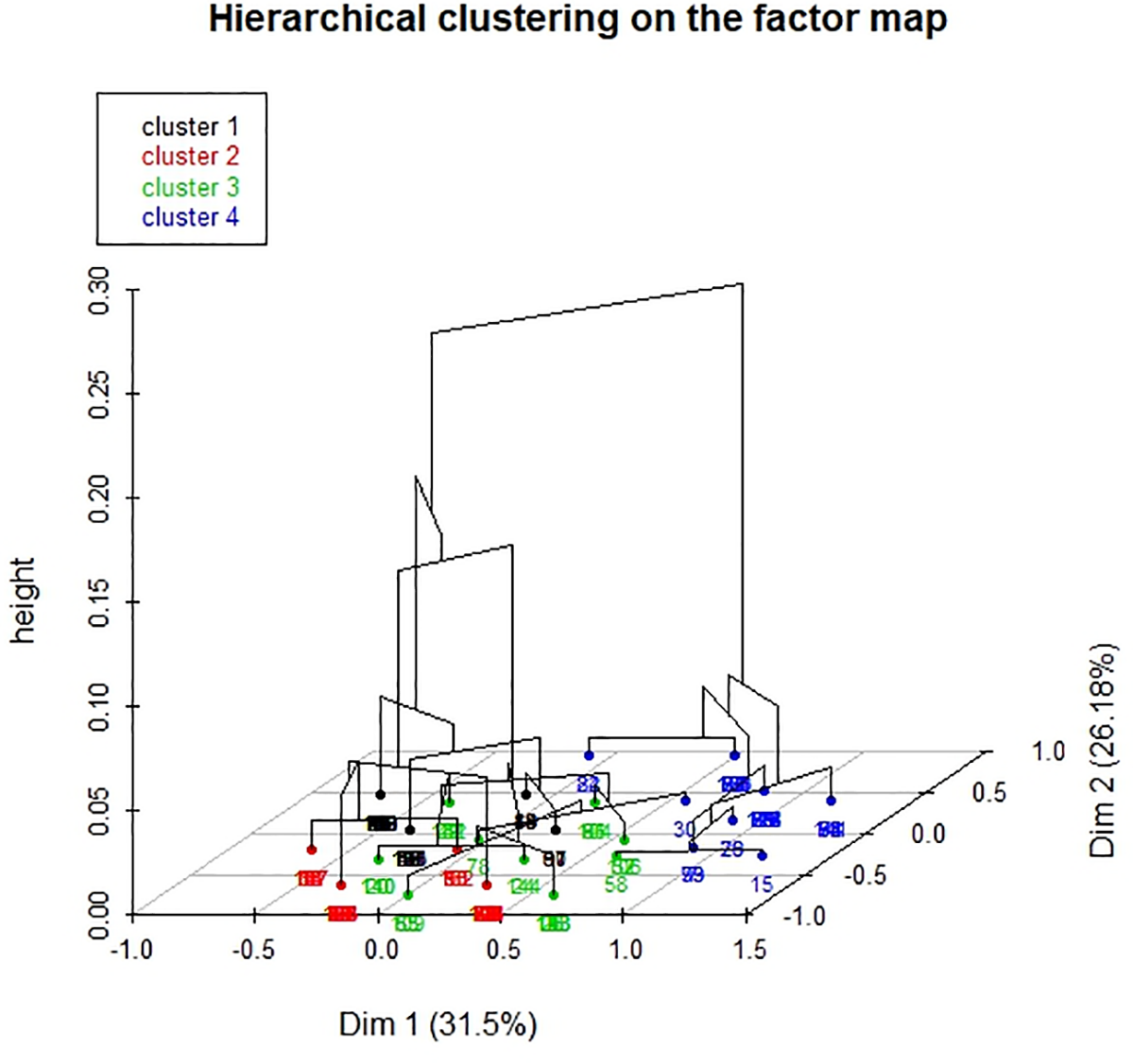
Hierarchical ascending classification of patients for dimensions 1 and 2. A three-dimensional hierarchical tree and a factorial plane in which individuals are colored according to the cluster to which they belong. The classification of individuals yielded four clusters.

The hierarchical ascendant classification of individuals yielded four clusters, as shown in Figs 3 and 4. The first cluster groups together individuals with a frequent absence of major events (sepsis, major bleeding, thrombotic event, major transfusion, renal replacement therapy, death; in decreasing order of frequency). The second cluster groups together individuals with a frequent presence of sepsis, a frequent absence of thrombotic event, and a frequent absence of major transfusion. The third cluster is characterized by a high frequency of thrombotic event and a frequent absence of major transfusion. Individuals in the last cluster had a frequent presence of major transfusion, a presence of major bleeding, and an absence of sepsis.

## Discussion

To the best of our knowledge, this study is the first to analyze the relationship between complications and between these and death in patients undergoing ECMO. Previous studies have focused on one complication at a time (nosocomial infection, thromboembolic event, bleeding, transfusion, etc.) and on the relationship between this specific complication and death [1–3, 8, 9, 23, 24]. Our study used a large cohort and a methodology that allowed us to highlight: *i*) complications that cluster together and cluster close to death; and *ii*) four patterns of patients according to complications, including one with no complications.

The mortality rate in our cohort of patients undergoing VA-ECMO was high at 50.3%. The rate of renal replacement therapy was 57.2%, the rate of sepsis was 44.8%, and the rate of major bleeding was 47.6%. In the meta-analysis on VA-ECMO published by Xie *et al*., the mortality rate at 30 days varied from 47.5% for cardiogenic shock to 64% for cardiac arrest [2]. This same meta-analysis reported a rate of kidney injury of 47.4%, a rate of major bleeding of 25.8%, and a rate of infection of 25.1%. Schmidt *et al*. reported a rate of nosocomial infection of 64% for their 220 patients under VA-ECMO, which is close to our findings [6]. The rate of major bleeding was higher in our cohort, which may be partly explained by the definitions we used. Unlike the many studies that classify complications according to organs (for instance, by counting intracranial bleeding as a neurological complication), we chose to classify them according to physiopathological mechanisms (for example, by grouping ischemic stroke and mesenteric infarction together). Lastly, our study revealed a high multi-morbidity rate, with around one third of patients presenting three or more of the five complications studied (*i*.*e*., sepsis, major transfusion, thrombotic event, major bleeding, and renal replacement therapy). To our knowledge, these data are new and cannot be compared to those published in other studies.

These high mortality and morbidity rates justify our global analysis of the multidirectional links between complications, which likely form a vicious circle leading to patient death. Indeed, we identified several interesting patterns of patients according to complications: The first pattern includes patients who had no complications and survived. The second and third patterns include patients with isolated sepsis or with an isolated thrombotic event. The last pattern reveals an interesting association between major transfusion, major bleeding, and an absence of sepsis. The first pattern of patients reinforces everyday perceptions, according to which some patients end up presenting several complications and even dying while others develop almost no complications. We excluded from our analysis patients who underwent ECMO for a very short period of time (less than two days) precisely in order to limit potential bias resulting from bad indication of ECMO—for instance, in moribund patients or, on the contrary, in patients who did not need ECMO and could have been located in cluster 1. While the complications “major bleeding” and “major transfusion” could have been coded under the same variable, we chose to distinguish between them for two reasons: *i*) patients can present major bleeding without receiving transfusion (for example, in the case of cerebral hemorrhage); and *ii*) transfusion is sometimes performed in the absence of major bleeding, especially in the presence of hemolysis.

Our study has several limitations. First, given its retrospective nature, bias may have been introduced, especially in the definition of the complications studied. Second, the monocentric character of the study may limit extrapolation of our findings to other ICUs. This, however, is a minor limitation, since we do not advance formal and precise conclusions but propose a broad and general approach—one that has been used in other contexts and that could inspire further research [13–16]. Third, our study focuses on complications in patients undergoing ECMO, and not on complications specifically related to ECMO, which may limit the ability to prevent ECMO-related complications. Nevertheless, it remains of interest to study and understand complications such as sepsis and renal failure, which can occur independently of ECMO.

In conclusion, our study, based on a large cohort of patients undergoing VA-ECMO in ICU and presenting a mortality rate comparable to that reported in the literature, identified numerous and often interrelated complications. The multiple correspondence analysis and the hierarchical ascendant classification yielded clusters of patients, while highlighting specific links between some of the complications studied. Further research should be conducted in this area.

## Supporting information

S1 Table Dataset. Patient's data.
(CSV)
